# Exercise in mice ameliorates high-fat diet-induced nonalcoholic fatty liver disease by lowering HMGCS2

**DOI:** 10.18632/aging.202717

**Published:** 2021-03-01

**Authors:** Xiaoli Qian, Ting Wang, Jiahong Gong, Li Wang, Xuyan Chen, Haiyan Lin, Wenzhan Tu, Songhe Jiang, Shengcun Li

**Affiliations:** 1 Rehabilitation Medicine Center, The Second Affiliated Hospital and Yuying Children’s Hospital of Wenzhou Medical University, Wenzhou 325027, Zhejiang, China; 2 Integrative and Optimized Medicine Research Center, China-USA Institute for Acupuncture and Rehabilitation, Wenzhou Medical University, Wenzhou 325027, Zhejiang, China

**Keywords:** exercise, NAFLD, high-fat diet, HMGCS2, Wnt3a/β-catenin

## Abstract

Nonalcoholic fatty liver disease (NAFLD) is a common chronic liver disease worldwide. Exercise is a therapeutic strategy for preventing NAFLD. However, the underlying molecular mechanisms by which NAFLD can be ameliorated through exercise are still not clear. This study investigates the mechanisms by which exercise suppresses NAFLD development induced by a high-fat diet (HFD) in mice. Male 6-week-old C57BL/6J mice were fed a normal diet or HFD for 12 weeks and then induced to swim or remain sedentary for 8 weeks. Histomorphology, inflammatory factors, fat metabolizing enzymes, fibrosis, and steatosis were determined in HFD-fed mouse liver, and levels of hepatic enzymes and molecules in the related pathways were analyzed. NAFLD mice showed evident steatosis, fibrosis, and liver injury, and an increased expression of HMGCS2, Wnt3a/ β-catenin, and phosphorylated (p)-AMPK in the liver. Exercise significantly attenuated these symptoms and downregulated the level of Wnt3a/β-catenin in lipotoxic liver tissue. Inhibition of HMGCS2 expression decreased the activation of the Wnt3a/β-catenin pathway and lowered p-AMPK in palmitate-treated HepG2. Our results suggest that exercise prevents NAFLD-associated liver injury, steatosis, and fibrosis. Exercise-mediated hepatoprotection was achieved partly via the blocking of the upregulation of HMGCS2 and the attenuation of the Wnt3a/β-catenin pathway.

## INTRODUCTION

Nonalcoholic fatty liver disease (NAFLD) is defined as a chronic liver disease characterized by fat accumulation exceeding 5.5% of the liver wet weight, which is not caused by alcohol consumption [1]. Because of the close association of NAFLD with type 2 diabetes mellitus and obesity, the prevalence of NAFLD is increasing worldwide [2, 3]. In fact, 25% of the world's population is currently thought to have NAFLD [4], causing a tremendous clinical and economic burden and poor health-related quality of life.

However, there are no pharmacological agents readily available for the specific treatment of NAFLD. The prevailing theory of NAFLD pathogenesis is based on the double-hit hypothesis. Insulin resistance and abnormal hepatocellular lipid accumulation represent the first hit [5], which in turn triggers a second hit, such as oxidative stress, inflammation, apoptosis and fibrosis, leading to the occurrence of nonalcoholic steatohepatitis. The current methods for treating NAFLD include exercise, a rational diet and medicines that treat some of the metabolic symptoms (fibrates, statins, and metformin) [6, 7]. As a nonpharmacological means, regular exercise can be considered an effective treatment strategy for the first hit of NAFLD [8]. Exercise improves NAFLD by reducing hepatic fat accumulation [9], increasing β-oxidation of fatty acids, inducing hepatoprotective autophagy, attenuating glucose control, and improving insulin sensitivity [10]. Additionally, exercise training suppresses the overproduction of reactive oxygen species and the upregulation of several antioxidant enzymes and anti-inflammatory mediators [8, 11]. Thus, a comprehensive understanding of the effects of exercise and the molecules involved in the associated signaling pathways may provide valuable insights into the progression of NAFLD and the methods for the development of suitable clinical therapies or novel drugs.

Hepatic lipid accumulation promotes systemic metabolic dysfunction [12], such as hepatic non-esterified fatty acid upregulating expression of genes involved in gluconeogenesis [13], beta-oxidation, lipogenesis, and ketogenesis, thereby promoting hyperglycemia, hyperlipidemia, and ketonemia [14]. Ketogenesis occurs mainly in hepatic tissues, and 3-hydroxy-3-methylglutaryl-CoA synthase 2 (HMGCS2) is the rate-controlling enzyme for ketone body synthesis during states of high fatty acid availability and oxidation [15]. Recent findings suggest that exercise-mediated cardioprotection through upregulation of miR-344g-5p, which targets *Hmgcs2* mRNA, blocks upregulation of HMGCS2 and thus protects against HFD-induced cardiomyopathy [16]. Therefore, we suspected that HMGCS2 is associated with NAFLD liver injury under lipotoxic conditions.

In this study, we explored the mechanisms by which exercise can prevent NAFLD, which may provide a rational research basis for the development of new drugs. In addition, exercise can be mimicked by new drugs, which could be beneficial for people who are clinically unable to exercise, such as those who become overweight or have a disability. We established a mouse model for diet-induced NAFLD with fatty liver symptoms similar to human metabolic syndrome. Our data indicate that exercise attenuated diet-induced hepatic injury, inflammation, fibrosis, and apoptosis. Inhibition of HMGCS2 induced by exercise might usefully contribute to hepatic protection in NAFLD. Exercise can prevent and treat various chronic metabolic diseases. Our study may have a translational value of mice NAFLD to human disease.

## RESULTS

### Exercise decreased body weight, liver weight, and fat content in HFD-fed mice

**Figure 1:**
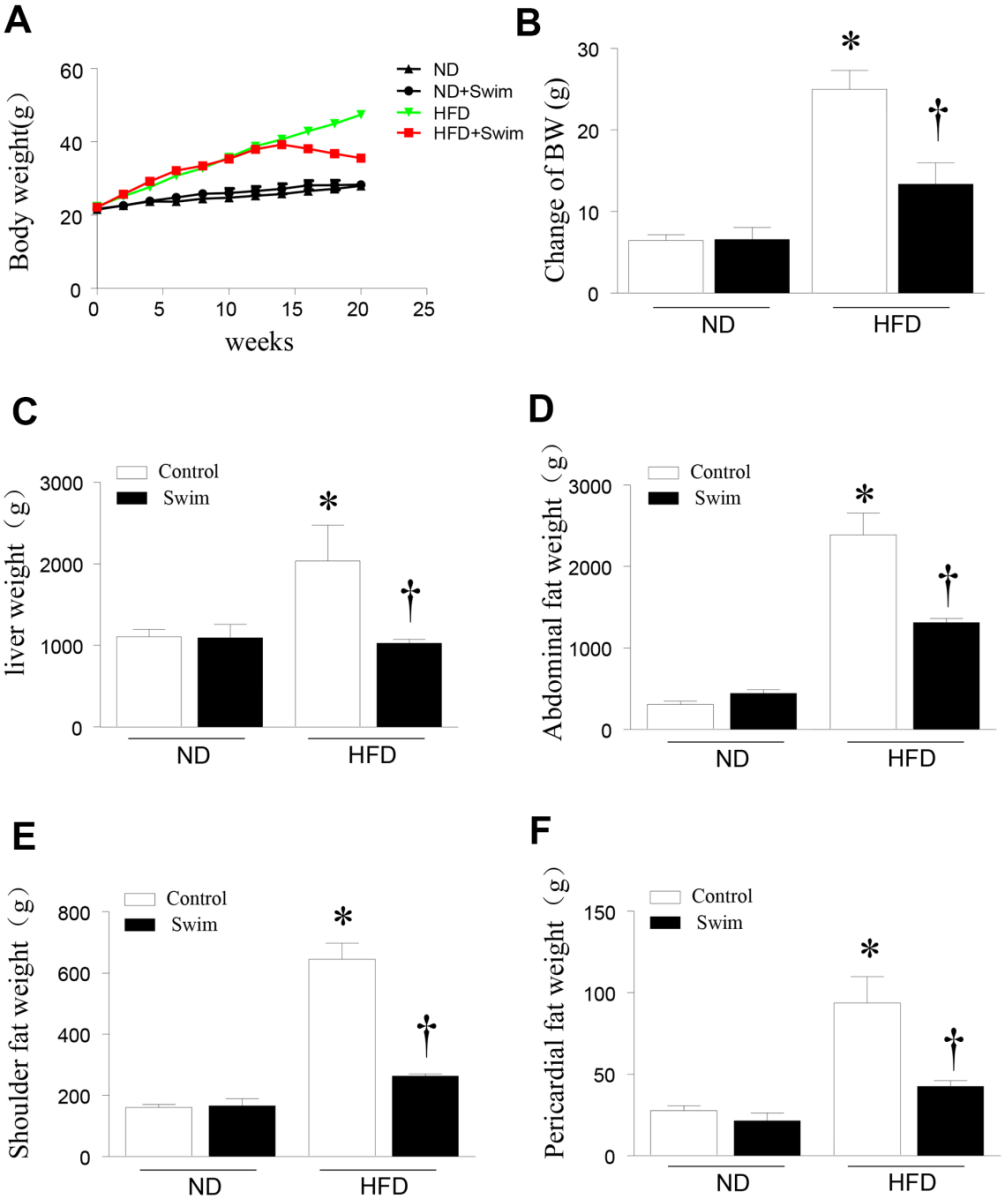
**Exercise reduced liver weight and fat content in HFD-fed mice.** (**A**) Body weight, (**B**) change in body weight, (**C**) weight of liver, (**D**) abdominal fat, (**E**) scapular fat, and (**F**) pericardial fat in mice. Data are presented as mean ± SD, n = 6. * P < 0.05 vs ND+SED and † P < 0.05 vs HFD+SED.

To investigate the role of exercise in NAFLD, we fed C57/BL6 mice normal diet (ND) or HFD to establish a model of NAFLD mice. The mice then remained sedentary or performed exercise in the form of swimming for 8 weeks. Twenty weeks after HFD feeding, mice exhibited an increased body weight (Figure 1A, 1B) and liver weight (Figure 1C) compared to the ND-fed group. The HFD significantly increased the abdominal fat weight (Figure 1D), shoulder fat weight (Figure 1E), and pericardial fat weight (Figure 1F). All parameters were ameliorated in NAFLD mice after 8 weeks of swimming exercise.

### Exercise improved metabolic parameters and insulin sensitivity in HFD-fed mice

**Figure 2:**
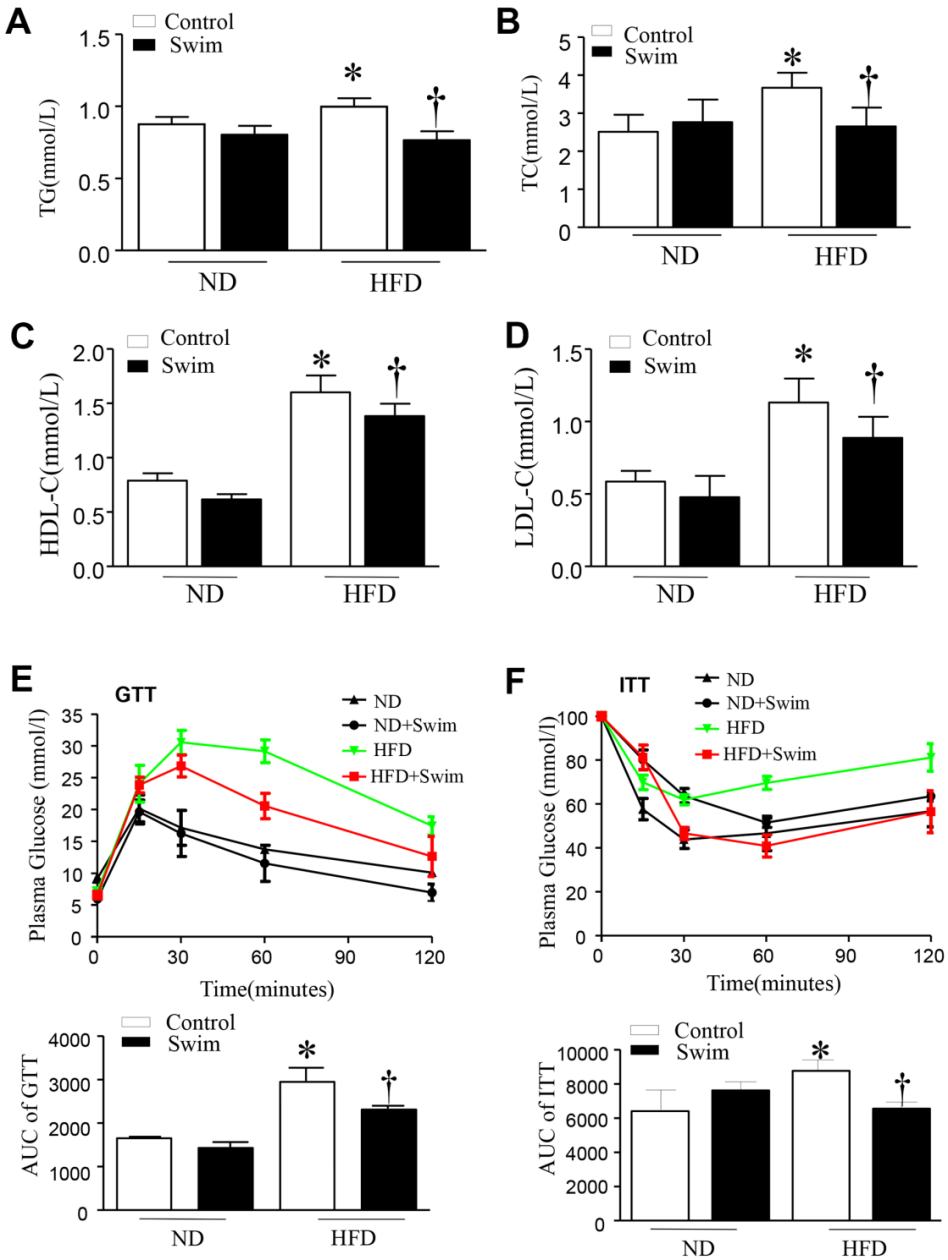
**Exercise ameliorated biochemical parameters in the serum of HFD-fed mice.** (**A**) Serum triglyceride (TG), (**B**) total cholesterol (TC), (**D**) low density lipoprotein (LDL) and (**C**) high density lipoprotein (HDL) levels, (**E**) Glucose tolerance test with area under the curve (AUC), (**F**) Insulin tolerance test with AUC. Data are presented as mean ± SD, n = 6. * P < 0.05 vs ND+SED and † P < 0.05 vs HFD+SED.

After twenty weeks of HFD, mice exhibited hyperlipidemia, with elevated total cholesterol (TC), triglyceride concentration (TG), LDL-C, and HDL-C in serum (Figure 2A–2D). They also displayed impairment in glucose tolerance tests (GTT) and insulin resistance tests (ITT) (Figure 2E, 2F) compared to the ND-fed group. All parameters were ameliorated in HFD mice with 8 weeks of swimming exercise.

### Exercise improves liver fat, pathomorphology, and hepatic injury in mice fed an HFD

**Figure 3:**
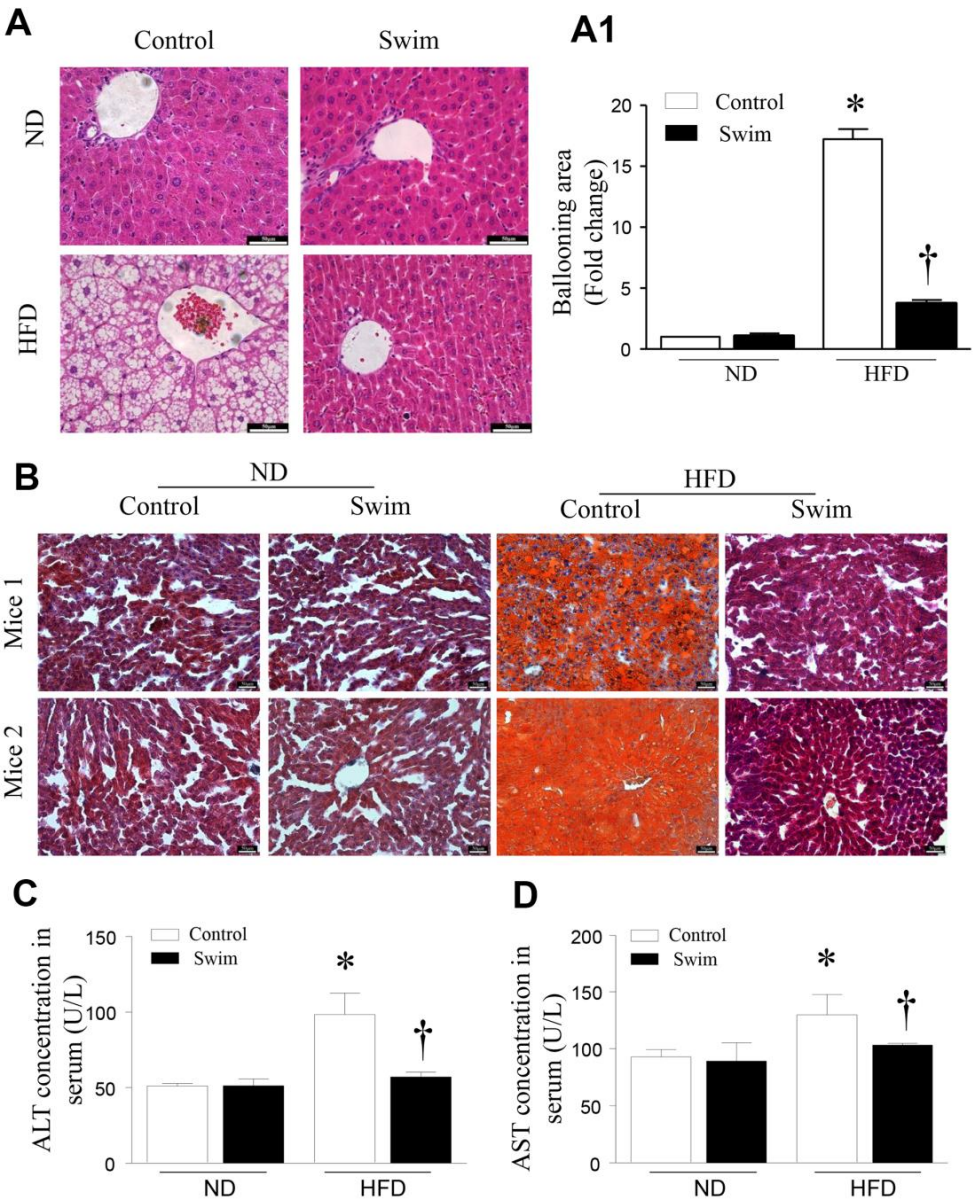
**Exercise ameliorated liver histological changes and steatosis.** (**A**) Hepatic steatosis and ballooning area (**A1**) determined by H&E and (**B**) Oil Red O staining for hepatic fat (scale: 50 μm). (**C**, **D**) The serum levels of ALT and AST in mice induced by HFD. Data are presented as mean ± SD, n = 6. * P < 0.05 vs ND+SED and † P < 0.05 vs HFD+SED.

As expected, animals receiving the HFD developed liver pathology typical of NAFLD. Histological analysis of hematoxylin-eosin (H&E)-stained revealed hepatic steatosis in both liver tissue and macrovesicular (Figure 3A, 3A1) from the NAFLD group. Likewise, we found that the livers of HFD-fed mice showed numerous small lipid droplets (Figure 3B). As shown in Figure 3, HFD-induced liver histological changes and lipid accumulation could be prevented by swimming exercise, which had a protective effect on liver steatosis.

Steatosis of the liver leads to decreased liver function, and increased serum alanine aminotransferase (ALT) and aspartate aminotransferase (AST) activities, which are indicative of hepatocyte injury. We found that the levels of serum ALT (Figure 3C) and AST (Figure 3D) were significantly higher in HFD-fed mice than corresponding levels in the ND group; however, these were reduced after 8 weeks of swimming in HFD-fed mice.

### Exercise prevents inflammation, de novo lipogenesis, and fibrosis in NAFLD mice

**Figure 4:**
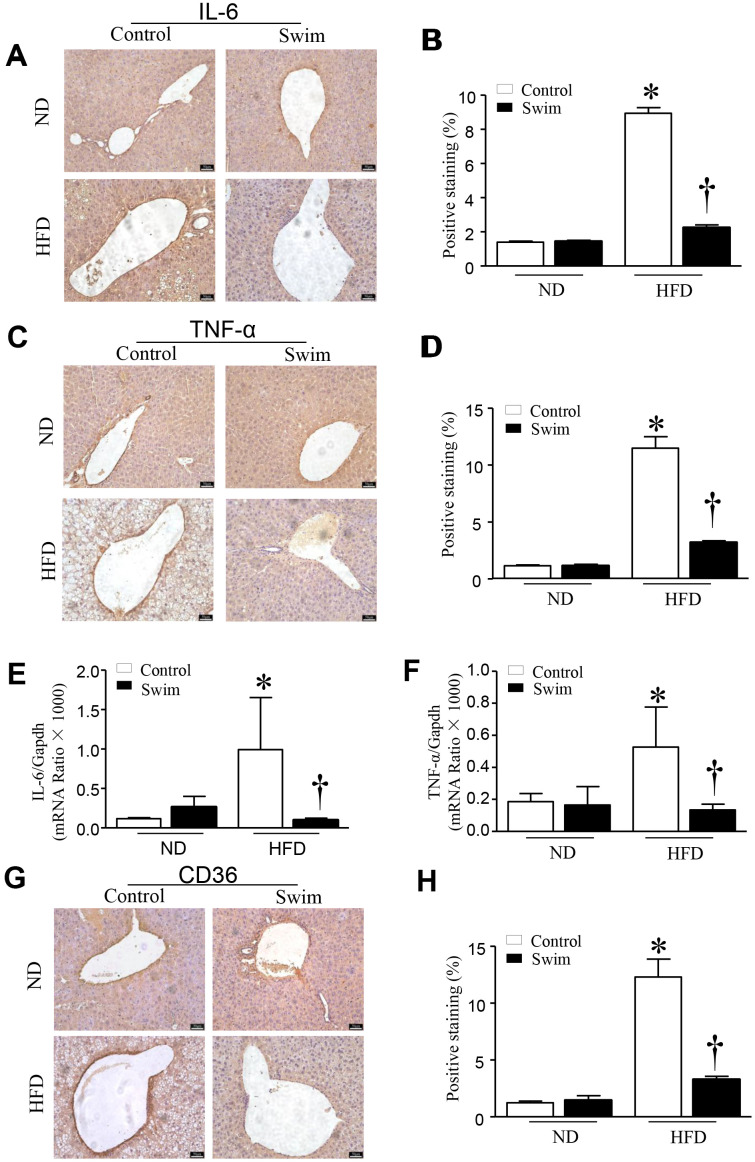
**Exercise reduces inflammatory indices in HFD-fed mice.** Representative and quantification with immunohistochemical staining of IL-6 (**A**, **B**), TNF-α (**C**, **D**) and CD36 (**G**, **H**) in the liver tissue. Quantification of mRNA for IL-6 (**E**), and TNF-α (**F**). Data are presented as mean ± SD, n = 6. * P < 0.05 vs ND+SED and † P < 0.05 vs HFD+SED.

HFD-fed mouse liver showed increased protein levels of both IL-6 (Figure 4A, 4B) and TNF-α (Figure 4C, 4D), and an increase in the mRNA levels of IL-6 (Figure 4E) and TNF-α (Figure 4F), providing further insights into the biochemistry underlying the second hit of NAFLD.

**Figure 5:**
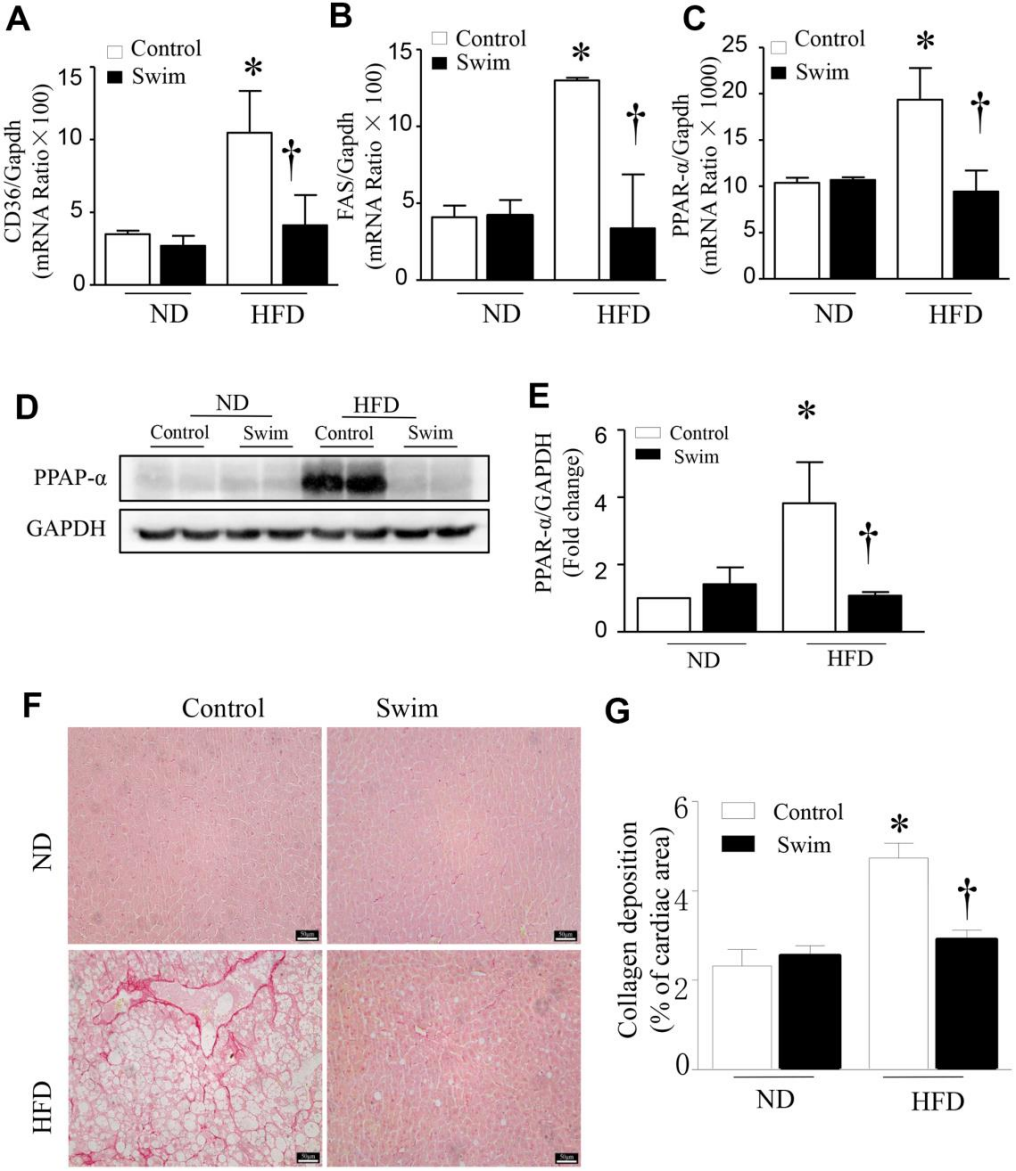
**Exercise prevented hepatic injury and fibrosis in HFD-fed mice.** Quantification of mRNA for (**A**) CD36, (**B**) FAS, and (**C**) PPAR-α. (**D**) Representative western blot for PPAR-α from at least 3 different cultures, (**E**) Quantification of PPAR-αprotein levels relative to GAPDH. (**F**) Representative picrosirius red staining for collagen deposition (red color). (**G**) Quantification for total collagen deposition. Data are presented as mean ± SD, n = 6. * P < 0.05 vs ND+SED and † P < 0.05 vs HFD+SED.

Furthermore, we found that the fatty acid translocase (FAT)/CD36 (Figures 4G, 4H, 5A), and genes involved in fatty acid synthesis (FAS) (Figure 5B) and fatty acid β-oxidation (proliferator-activated nuclear receptor, PPAR-α) (Figure 5C–5E) were significantly upregulated in the liver of NAFLD mice, whereas they were lowered after swimming exercise. These results indicated that though large amounts of lipid droplets and increased fatty acids levels were present in the liver (Figure 3B), very active fatty acid synthesis and catabolism still occurred. These results also suggested that de novo lipogenesis was still occurring in NAFLD liver tissue, despite a systemic overabundance of fat.

Liver fibrosis is closely related to the progression of NAFLD. To determine tissue fibrosis, picrosirius red staining was used to visualize collagen. As shown in Figure 5F, 5G, the deposition of total collagen increased in the livers of HFD-fed mice. Simultaneously, inflammation, de novo lipogenesis, and collagen deposition in the liver decreased significantly after 8 weeks of swimming.

### Exercise regulated HMGCS2 and Wnt3a/β-catenin in lipotoxic conditions

**Figure 6:**
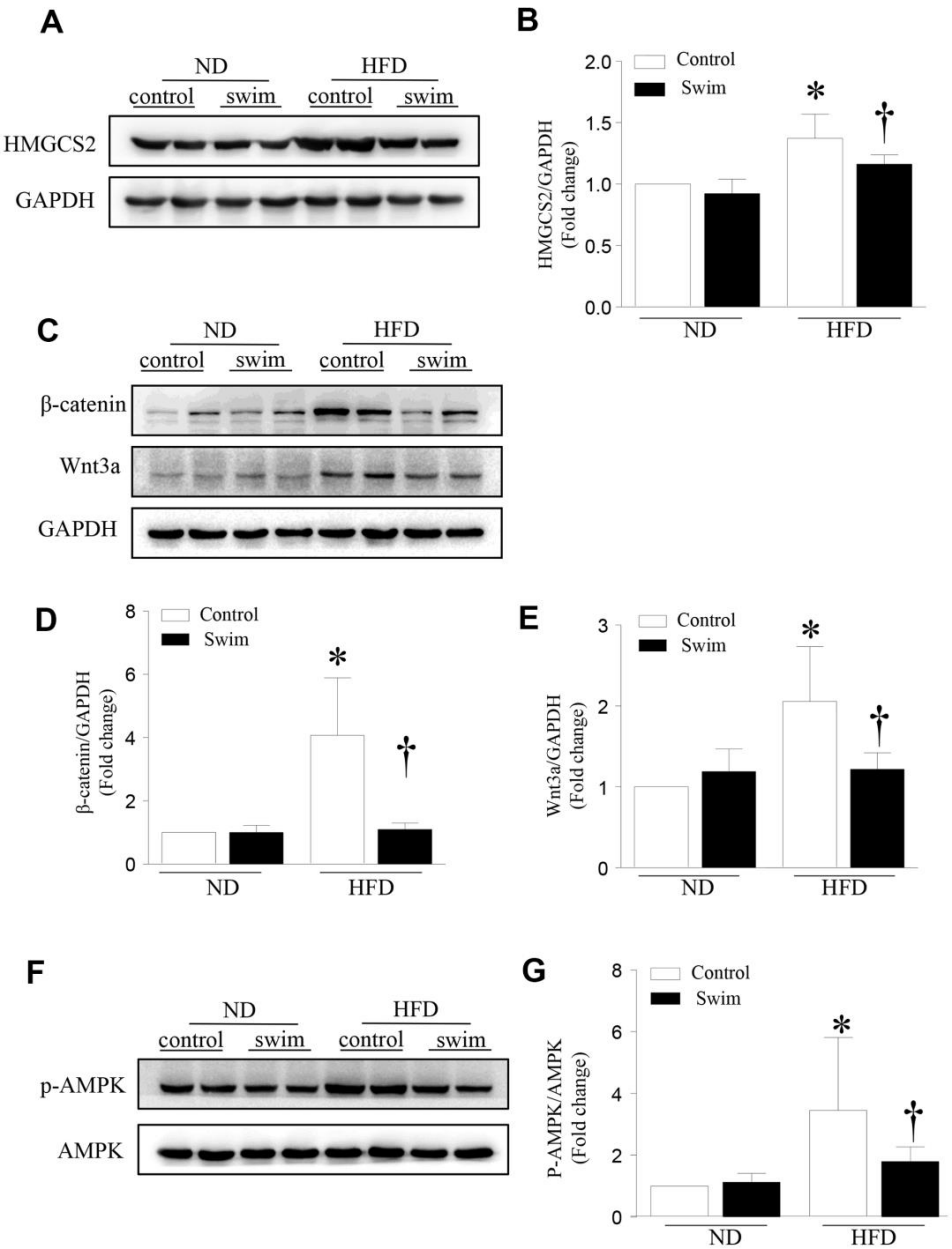
**Exercise-dependent reduction of HFD-induced expression of HMGCS2 and Wnt3a/β-catenin in hepatic tissue.** (**A**) Representative western blot for HMGCS2 in mouse liver. (**B**) Quantification of HMGCS2 protein levels relative to GAPDH. (**C**) Representative western blot for Wnt3a/β-catenin in mouse liver. (**D**, **E**) Quantification of Wnt3a and β-catenin protein levels relative to GAPDH. (**F**) Representative western blot for p-AMPK in mouse liver. (**G**) Quantification of p-AMPK protein levels relative to AMPK. Data are presented as mean ± SD, n = 6. * P < 0.05 vs ND+SED and † P < 0.05 vs HFD+SED.

HMGCS2 is broadly distributed in liver tissue and plays an important role in regulating tissue growth and metabolism. Previous studies have found that HMGCS2 mRNA and protein expression increased in mouse heart tissue after HFD intake [16]. However, the direct relationship between the expression of HMGCS2 and NAFLD is not clear. Therefore, we assessed the expression level of HMGCS2 in mice through western blotting (Figure 6A, 6B). Our results indicated that in HFD-fed mice, HMGCS2 increased in the liver but these levels decreased after exercise. Similarly, exercise lowered the levels of Wnt3a and β-catenin in the hepatic tissue of HFD-fed mice (Figure 6C–6E). In addition, exercise significantly lowered the levels of p-AMPK (Figure 6F, 6G) in lipotoxic hepatic tissue.

### HMGCS2 regulates the Wnt3a/β-catenin pathway

**Figure 7:**
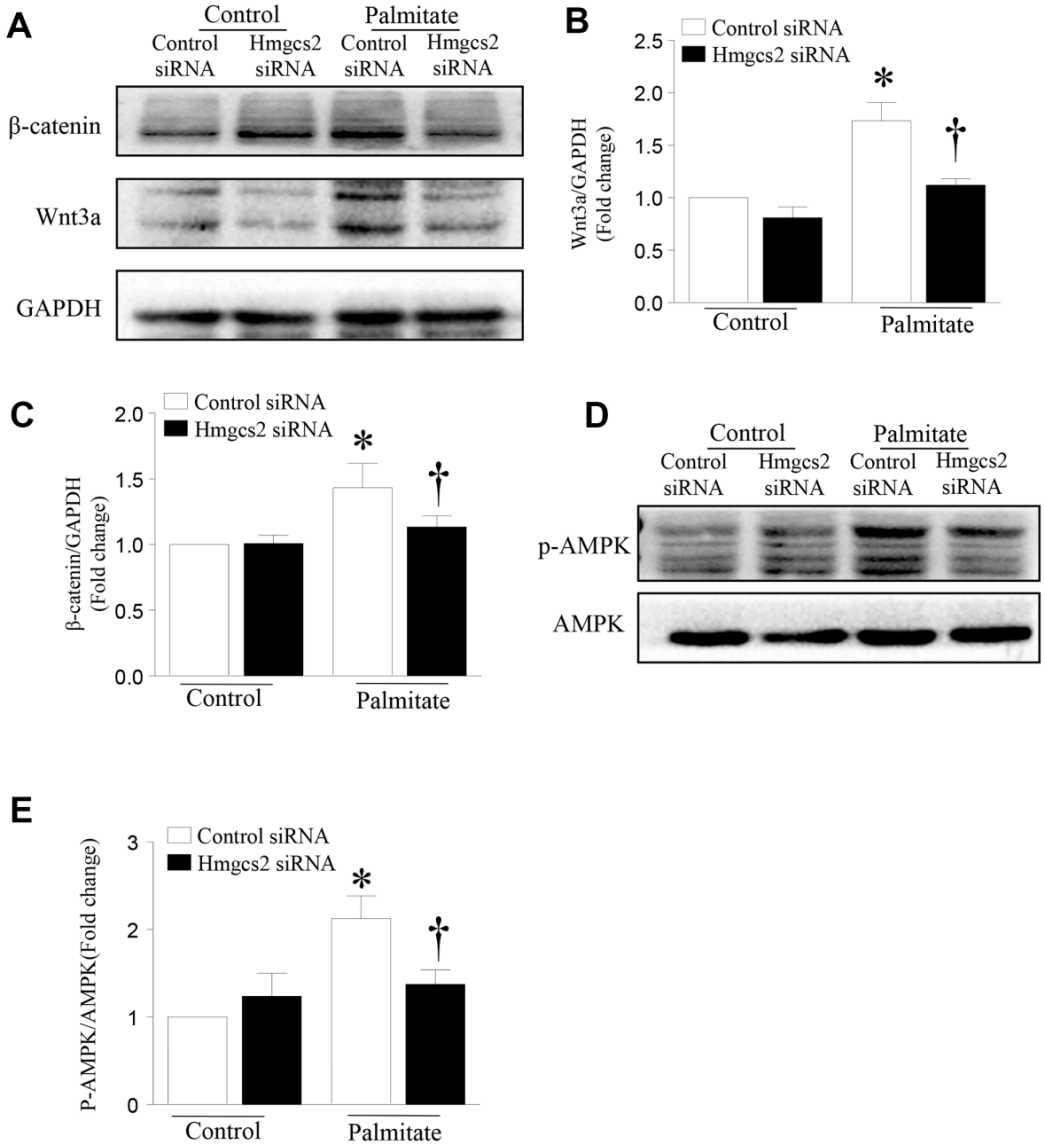
**HMGCS2 regulates Wnt3a/β-catenin signal transduction and AMPK phosphorylation in HepG2 cells.** Cultured HepG2 cells were transfected with *Hmgcs2* siRNA or control siRNA, followed by incubation with high palmitate or oleate for 24 h. (**A**) Representative western blot for Wnt3a/β-catenin. (**B**, **C**) Quantification of Wnt3a/β-catenin protein levels relative to GAPDH from three different cultures. (**D**) Representative western blot for p-AMPK in mouse liver. (**E**) Quantification of p-AMPK protein levels relative to total AMPK. Data are presented as mean ± SD, n = 3. * P < 0.05 vs control + control siRNA and † *P* < 0.05 vs palmitate + control siRNA.

To test whether HMGCS2 can regulate the Wnt3a/β-catenin pathway, we used *Hmgcs2* siRNA to inhibit its expression. HepG2 cells were transfected with *Hmgcs2* siRNA for 24 h and then incubated with palmitic acid for another 24 h. As shown in Figure 7, the levels of Wnt3a and β-catenin were significantly decreased after inhibition of HMGCS2 in palmitic acid-induced HepG2 cells. Similarly, the activity of AMPK decreased with the inhibition of HMGCS2 under lipotoxic conditions. These results suggest that the inhibition of HMGCS2 expression can reduce the expression of Wnt3a/β-catenin and the phosphorylation of AMPK.

### Inhibition of HMGCS2 decreases lipotoxicity-induced apoptosis

**Figure 8:**
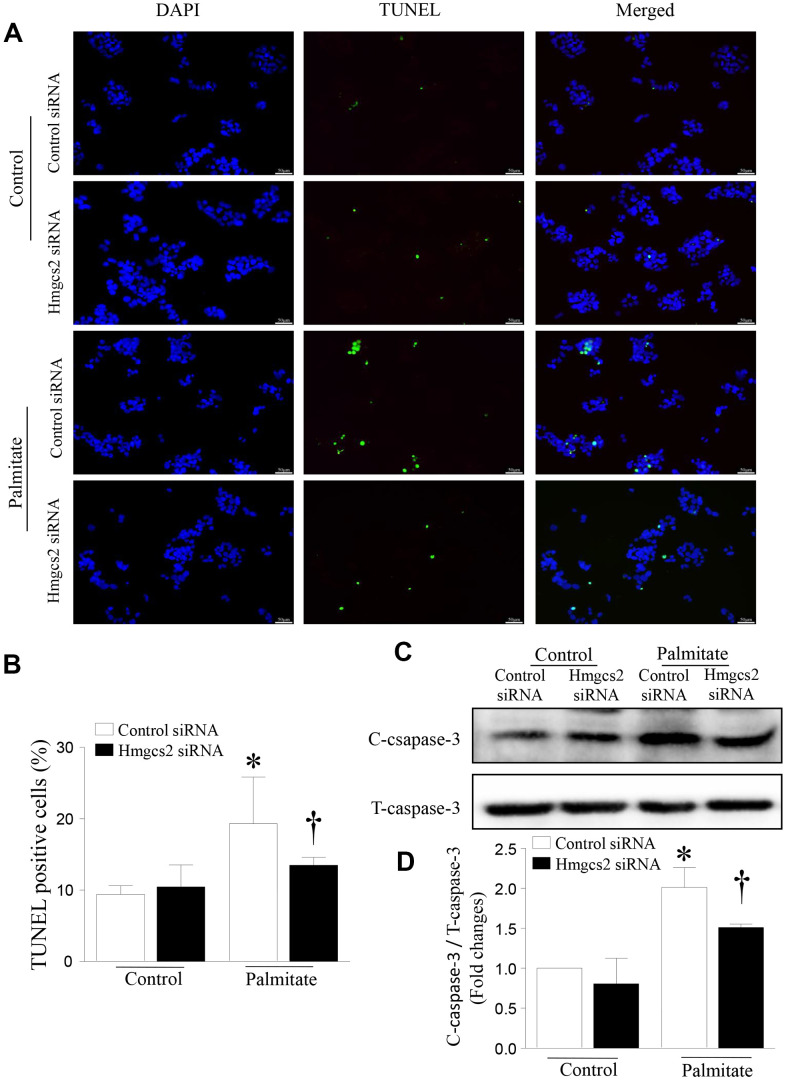
**HMGCS2 regulated HepG2 apoptosis in lipotoxic conditions.** Cell apoptosis was assessed by TUNEL staining (green), and the nucleus was stained using propidium iodide (PI) (blue). (**A**) Representative images of TUNEL staining. (**B**) Quantification of TUNEL-positive cells from three different independent cultures. (**C**) Representative western blot for cleaved caspase-3 and total caspase-3. (**D**) Quantification of cleaved caspase-3 protein levels relative to total caspase-3 from three different cultures. Data are presented as mean ± SD, n = 3. * *P* < 0.05 vs control + control siRNA and † *P* < 0.05 vs palmitate + control siRNA.

To determine the effect of HMGCS2 on the viability of hepatic cells, we analyzed apoptosis of HepG2 cells incubated with palmitic acid and *Hmgcs2* siRNA. As shown in Figure 8, *Hmgcs2* siRNA decreased palmitate-induced hepatic apoptosis; this was evident from the reduced number of TUNEL-positive cells (Figure 8A, 8B) and lower levels of cleaved caspase-3 (Figure 8C, 8D).

## DISCUSSION

NAFLD is an important precipitating factor for steatohepatitis and fatty liver disease. As there is currently no accepted pharmacological treatment directly targeting NAFLD, lifestyle interventions are the major treatment options, including exercise [17], dietary strategies [18], and herbal and other non-specific medicines [19]. As a nonpharmacological means, physical exercise can not only change the course of systemic metabolic syndrome but also promote skeletal muscles to secrete immune factors and improve hepatic pathology in NAFLD. However, our understanding of the mechanisms by which exercise induces molecular changes in liver tissue is limited. This study demonstrated that 8 weeks of exercise prevented lipotoxicity-induced hepatic injury, inflammation, and steatosis by inhibiting HMGCS2, subsequently ameliorating apoptosis and normalizing the Wnt3a/β-catenin pathway.

Consistent with our results, in another randomized controlled trial, 12 weeks of exercise significantly reduced hepatic triglyceride content, visceral fat, and plasma triglyceride levels in patients with NAFLD [20]. Both aerobic and resistance exercise of similar frequency, duration, and periods (40–45 min/session, 3 times/week for 12 weeks) were found to reduce hepatic steatosis in NAFLD patients; however, intensity and energy consumption was significantly lower for resistance exercise than for aerobic exercise [21]. In addition, endurance exercise attenuated hepatic cholesterol overload and the ensuing severe oxidative stress in diet-induced NAFLD [22].

Although the protective effects of exercise on NAFLD are well known, it is not clear whether the effects on ketone metabolism are replicated in the liver. We have recently demonstrated the upregulation of the rate-limiting ketogenic enzyme HMGCS2 in the hearts of HFD-fed mice, whereas the inhibition of HMGCS2 acts to protect cardiomyocytes [16]. Similarly, our results suggest that exercise reduces HFD-induced HMGCS2 expression in the liver. Ketogenesis can dispose of much of the fat that enters the liver, and dysfunction in this pathway can promote the development of NAFLD. HFD-fed mice with insufficient ketogenesis display extensive hepatocyte injury and inflammation, decreased glycemia, an abnormal concentration of hepatic tricarboxylic acid (TCA) cycle intermediates, and impaired hepatic gluconeogenesis due to the sequestration of free coenzyme A (CoASH) [23]. Thus, it appears that ketogenesis is beneficial for the prevention of fatty liver disease. However, the pathogenic process of NAFLD is strongly linked to overnutrition, and lipotoxicity promotes the metabolism of active ketone bodies, accompanied by an increased HMGCS2 activity [24]. High energy anabolism, such as that induced by hunger [25] and lack of insulin, has been shown to increase ketone body production in the heart and liver [26]. Ketogenesis is progressively impaired as hepatic steatosis and glycemia worsens [27]. Conversely, the alternative pathway for acetyl-CoA metabolism, oxidation in the TCA cycle, was found to be upregulated in NAFLD as ketone production diminished and was positively correlated with gluconeogenesis rates and plasma glucose concentrations [27].

Inhibition of HMGCS2 by exercise may also be due to the decrease in the amount of free fatty acids entering the liver. This view is consistent with the results of a recent study, which reported that a reduction in the need for fatty acid metabolism or an increased supply of glucose can inhibit HMGCS2 and decrease the production of ketone bodies [28]. Indeed, in our *in vitro* study, inhibition of HMGCS2 by siRNA prevented palmitate-induced apoptosis in HepG2 cells. Collectively, these results suggest that in a mouse model of NAFLD, exercise exhibits a direct protective effect through the lowering of HMGCS2 expression.

Our study also found that the increased expression of HMGCS2 in the HFD-fed mice is associated with Wnt3a/β-catenin abundance in nonalcoholic fatty liver disease. Previous studies have demonstrated that the Wnt/β-catenin pathway is involved in a chain of molecular events in the livers affected by nonalcoholic fatty liver disease. Namodenoson exerts an anti-nonalcoholic steatohepatitis effect that is mediated by the deregulation of the PI3K/NFκB/Wnt/β-catenin signaling pathway [29, 30]. Hepatic miR-146a-5p was found to be downregulated in fibrosing steatohepatitis; however, its target genes, Wnt1 and Wnt5a, and their consequent effectors, α-SMA and Col-1, were significantly upregulated. Further, the overexpression of miR-146a-5p in hepatic stellate cells (HSC) inhibited HSC activation and proliferation, concomitant with a decreased expression of Wnt1, Wnt5a, α-SMA, and Col-1 [31]. Other reports have shown that exercise can inhibit the activation of the Wnt3a/β-catenin signaling pathway in the skeletal muscle of patients with type 2 diabetes, reducing Wnt3a protein levels and thereby reducing fat synthesis, improving lipid metabolism, and reducing muscle atrophy to ultimately improve insulin resistance [32]. In addition, Wnt3a significantly increased cellular caspase activities and TUNEL staining in response to hypoxia-reoxygenation [33], and siRNA against β-catenin markedly inhibited Wnt3a-activated caspase activation. Our results indicated that Wnt3a/β-catenin was reduced after HMGCS2 inhibition, and apoptosis was inhibited in palmitate-treated hepatocytes. HMGCS2 and WNT pathway activation increase was a common pathogenic factor for NAFLD. Therefore, we suspected that HMGCS2 was associated with wnt3a/β-catenin activation. Indeed, we found that the reduction of HMGCS2 due to exercise in animals, or the direct inhibition of HMGCS2 by siRNA, reduced the expression of both wnt3a and β-catenin. However, more detailed mechanisms need to be discussed in future studies.

Previous studies observed elevated AMPK activity in E4bp4-LKO female mice exhibiting reduced liver lipid accumulation and partially improved liver function after 10 weeks of HFD feeding [34]. Cardiotrophin-1 resolves hepatic steatosis in obese mice by mechanisms involving AMPK activation [35]. Contrary to these findings, other studies support our observation that the inhibition of AMPK by RNA interference or by use of inhibitors suppresses fatsioside A-induced caspase-3 cleavage and apoptosis in the HepG2 cells. In this study, under lipotoxic conditions, the activity of AMPK was increased, with effects that may be similar to those of an AMPK activator (AICAR). These include marked cytotoxic effects [35], such as restriction of protein synthesis, cell growth, and proliferation, which lead to increased apoptosis in HepG2 cells [36]. The damage and adverse outcomes caused by NAFLD were reversed by the inhibition of HMGCS2.

In summary, our study demonstrates that exercise in mice prevents NAFLD-associated liver injury, steatosis, liver fibrosis and inflammation, and de novo lipogenesis. Furthermore, exercise blocks HMGCS2 expression and restores normal levels of Wnt3a/β-catenin in lipotoxic liver tissue. We suggest that the HMGCS2/Wnt3a/β-catenin pathway is a novel mechanism associated with exercise-mediated liver protection against NAFLD. In the model of lipotoxic hepatocytes, the effect of exercise on protecting liver was mimicked by inhibiting HMGCS2. Exercise-reduced HMGCS2 related pathway may represent a potential target for the development of pharmacological interventions in human NAFLD.

## MATERIALS AND METHODS

### Animals and swimming protocol

The investigation with experimental animals conforms to the Guide for the Care and Use of Laboratory Animals published by the US National Institutes of Health (NIH Publication No. 85-23, revised 1996). C57BL/6 mice were purchased from the Model Animal Research Center of Wenzhou Medical University. Male mice (6-week-old) were housed at a constant temperature of 22° C in a 12-h light/dark cycle with free access to regular rodent chow and pure water.

The mice were allowed to acclimatize for at least 1 week. Thereafter, the animals were randomly divided into four groups: ND sedentary group (ND+SED), ND plus swimming exercise group (ND+SW), HFD sedentary group (HFD+SED), and HFD plus swimming exercise group (HFD+SW). Swimming exercise was started after 12 weeks of HFD. HFD continued for 20 weeks with or without 8 weeks of swimming exercise. The HFD consisted of 26.2% protein, 26.3% carbohydrate, and 34.9% fat (% by mass) (D12492, Research Diets Inc., New Brunswick, NJ, USA). The calculated caloric intake from these nutrients (% kcal) was 20%, 20%, and 60%, respectively. The ND consisted of 20% protein, 70% carbohydrate, and 10% fat (% by weight) (D12450B, Research Diets Inc., New Brunswick, NJ, USA). For the swimming exercise protocol, after 1 week of adaptive swimming, the time progressively increased every 3 d from 10 min to 45 min. Mice swam 5 d a week for 8 weeks.

### Metabolic parameters and insulin sensitivity test

The mice were euthanized at the end of the experiment. The serum was obtained by clotting the blood for 2 h at room temperature and centrifugation at 2000 *× g* for 15 min. The serum samples were collected into a sterile EP tube and stored at–80° C. The serum TG, TC, HDL-C, LDL-C, ALT, and AST levels were assayed using a kit (Nanjing Jiancheng Biology Engineering Institute, Nanjing, China).

For the glucose tolerance test (GTT), mice (16-h fasted) were administered a glucose load (2 g/kg, i.p.). For the insulin tolerance test (ITT), 5-h fasted mice were administered insulin (0.75 U/kg body weight). For both tests, blood samples were taken from the tail vein at 0, 15, 30, 60, and 120 min after injection, and blood glucose was measured using a glucose meter.

### Cell culture

HepG2 human hepatocellular carcinoma cells were purchased from Shanghai Fu Heng Biological Co., Ltd (Shanghai, China) and cultured in DMEM supplemented with 10% fetal bovine serum, 100 U/mL penicillin and 100 U/mL streptomycin. The cells were incubated at 37° C in a humid environment of 5% CO_2_ and 95% air.

### Oil red O staining

The liver tissue was fixed in 4% paraformaldehyde (PFA) for at least 24 h. Then, the tissue was embedded with Optimal Cutting Temperature and cut into 15-μm-thick sections. Finally, sections were stained with Oil Red O, and positively stained lipid droplets appeared red.

### H&E and sirius red staining

Livers were fixed in 4% PFA. Then, the tissue was embedded in paraffin and sectioned to 5-μm thickness. After the paraffin sections were dewaxed, morphological changes in the liver tissue were detected. H&E staining was used for pathological evaluation of the livers. Sirius red was used to detect liver fibrosis. A light microscope at 200× magnification (Leica, DM2500, Wetzlar, Germany) was used for capturing the photomicrographs.

### Immunohistochemistry and qRT-PCR

Immunohistochemistry and quantitative real time PCR (qRT-PCR) were performed as described previously [16].

### TUNEL staining

The TUNEL assay was performed to investigate the fragmentation of DNA during apoptosis. The cells were fixed with 4% PFA for 30 min. The slides were infiltrated with PBS solution containing 0.2% Triton-100 for 5 min at room temperature and then washed three times with buffer for 15 min. Apoptotic cells were detected using the DeadEnd™ Fluorometric TUNEL System (Promega, Madison, WI, USA). Images were visualized with a Leica microscope, and apoptosis-positive cells were counted.

### Western blotting

The prepared liver tissue samples were taken out from –80° C storage and lysed in protein lysis buffer containing phenylmethylsulfonyl fluoride (RIPA:PMSF = 100:1). The protein concentration was determined using the BCA kit (Beyotime Biotechnology, Shanghai, China). The same amount of total protein samples were subjected to 10% sodium dodecyl sulfate-polyacrylamide gel electrophoresis. The proteins were then transferred to polyvinylidene difluoride (PVDF) membranes (Millipore, Burlington, MA, USA). After blocking with 5% skimmed milk for 60 min, the blots were successively incubated with primary and secondary antibodies. Finally, protein expression and band intensity were detected using an ECL Western Blot detection kit (Thermo Fisher Scientific Inc., Rockford, USA). The primary antibodies used were anti-HMGCS2 (CST, 20940), -GAPDH (CST, 2118), -Wnt3a (CST, 2721), -β-catenin (CST, 8480), -p-AMPKα (1:1000, CST, 2531), and -caspase-3 (CST, 9662) at a concentration of 1:1000.

### Treatment of siRNA

Cholesterol-conjugated siRNAs for *Hmgcs2* were purchased from Shanghai GenePharma Co. Ltd (Shanghai, China). The sequences for *Hmgcs2* siRNA are as follows:

(sense 5′–3′) 5′-CCUCGAUGAUGUGCAGUAUTT-3′ and (antisense 5′–3′) 5′-AUACUGCACAUCAUCGAGGTT-3′. HepG2 cells were transfected with *Hmgcs2* siRNA or a control siRNA for 24 h followed by incubation with palmitate (300μmol/L) or control buffer for an additional 24 h.

### Statistical analysis

Data are expressed as mean ± SD of six animals. The level of statistical significance was determined using one-way analysis of variance. Student’s t-test was used for comparison between two groups. Statistical analyses were performed using GraphPad Prism v5.0 (GraphPad Software, San Diego, CA, USA), and p <0.05 indicated statistical significance.
